# Navigating the Light-Sheet Image Analysis Software Landscape: Concepts for Driving Cohesion From Data Acquisition to Analysis

**DOI:** 10.3389/fcell.2021.739079

**Published:** 2021-11-01

**Authors:** Holly C. Gibbs, Sakina M. Mota, Nathan A. Hart, Sun Won Min, Alex O. Vernino, Anna L. Pritchard, Anindito Sen, Stan Vitha, Sreeja Sarasamma, Avery L. McIntosh, Alvin T. Yeh, Arne C. Lekven, Dylan A. McCreedy, Kristen C. Maitland, Lisa M. Perez

**Affiliations:** ^1^Department of Biomedical Engineering, Texas A&M University, College Station, TX, United States; ^2^Microscopy and Imaging Center, Texas A&M University, College Station, TX, United States; ^3^Department of Biology, Texas A&M University, College Station, TX, United States; ^4^Department of Neurology, Baylor College of Medicine, Houston, TX, United States; ^5^Department of Biology and Biochemistry, University of Houston, Houston, TX, United States; ^6^High Performance Research Computing, Texas A&M University, College Station, TX, United States

**Keywords:** light-sheet, image analysis, parallel processing, multiview deconvolution, tool selection

## Abstract

From the combined perspective of biologists, microscope instrumentation developers, imaging core facility scientists, and high performance computing experts, we discuss the challenges faced when selecting imaging and analysis tools in the field of light-sheet microscopy. Our goal is to provide a contextual framework of basic computing concepts that cell and developmental biologists can refer to when mapping the peculiarities of different light-sheet data to specific existing computing environments and image analysis pipelines. We provide our perspective on efficient processes for tool selection and review current hardware and software commonly used in light-sheet image analysis, as well as discuss what ideal tools for the future may look like.

## Introduction

Since light-sheet microscopy was introduced to the life and biomedical science communities in 1993 (Voie et al., 1993) and more broadly in 2004 (Huisken et al., 2004), there has been a virtual Cambrian explosion of light-sheet instrumentation and image analysis tools [see here for recent reviews (Reynaud et al., 2015; Albert-Smet et al., 2019; Wan et al., 2019)]. Science and technology has always been a moving target, but the pace of light-sheet instrumentation and software development has been staggering. Researchers have adapted the basic light-sheet body plan to different applications with different lens geometries (Huisken and Stainier, 2007; Dunsby, 2009; Wu et al., 2011, 2013; Tomer et al., 2012; Kumar et al., 2014, 2018; Voleti et al., 2016, 2019; Sapoznik et al., 2020), beam shaping strategies (Keller et al., 2008; Planchon et al., 2011; Chen et al., 2014; Vettenburg et al., 2014; Liu et al., 2018; Chang et al., 2019), sample mounting and scanning techniques (Bouchard et al., 2015; Royer et al., 2016; Wu et al., 2017; Fadero et al., 2018; Glaser et al., 2019), and contrast mechanisms (Truong et al., 2011; Di Battista et al., 2019). The ability to image intact tissues, now made possible with advances in clearing protocols (Richardson and Lichtman, 2015; Matryba et al., 2019; Ueda et al., 2020; McCreedy et al., 2021), and also the desire to image naturally dynamic 3D biological systems with live-cell imaging are the two main forces driving this unusual technological variety. This variety stands in comparison to the more purely performance driven development of, for example, confocal microscopy where samples are typically uniformly thin layers, sections, or cell cultures on a slide.

**FIGURE 1:**
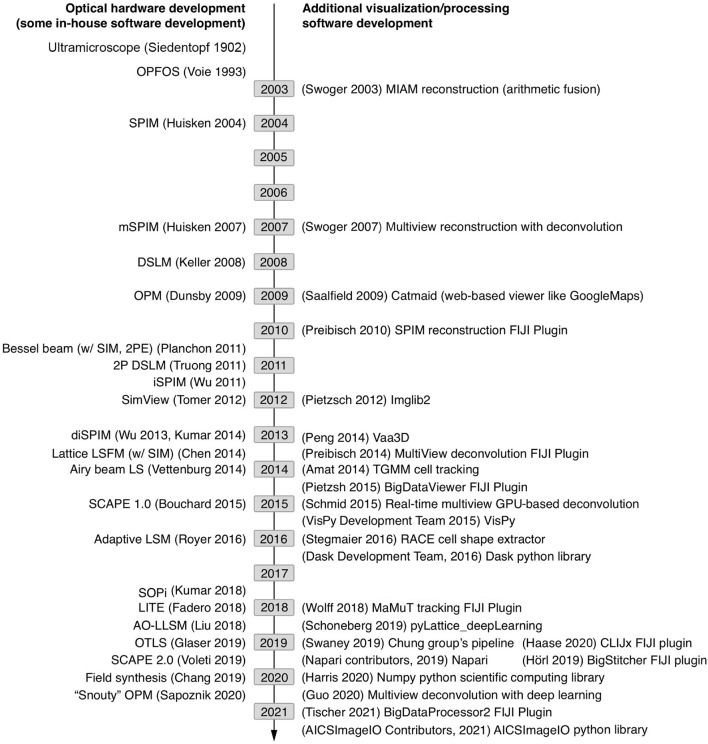
Timeline of sampled light-sheet microscopy optical hardware development and processing software across a variety of applications. As unique species of light-sheet microscopes have been developed, unique analysis solutions have been created.

All of these species of light-sheet microscopes result in large data acquisitions with unique, context-specific image processing considerations requiring savvy compression or computation strategies and often high-performance computing (HPC) hardware. Potential solutions in both the commercial and open-source software space employ a variety of strategies for managing the flow of data through a given image analysis pipeline. Light-sheet imaging hardware developments are overviewed in Figure 1 alongside the development of relevant software. Initially, microscope developers cobbled their own image analysis solutions together, typically made available upon request but not commonly actively maintained as they were iteratively improved. However, as broader interest in light-sheet microscopy increased, research groups employing or led by software developers have worked to make light-sheet image visualization and analysis tools more stable and accessible to biologists through web browser tools (Saalfeld et al., 2009), the java-based ImageJ/FIJI community (Preibisch et al., 2010, 2014; Pietzsch et al., 2012, 2015; Wolff et al., 2018; Hörl et al., 2019; Haase et al., 2020; Tischer et al., 2021), packaged C++ applications (Amat et al., 2014; Peng et al., 2014; Stegmaier et al., 2016), MATLAB code, and python libraries (Campagnola et al., 2015; Crist, 2016; Dask Development Team, 2016; Napari Contributors, 2019; Swaney et al., 2019; Harris et al., 2020; AICSImageIO Contributors, 2021). Likewise, instrumentation research groups have made light-sheet hardware more accessible through open-source DIY projects such as OpenSPIM (Pitrone et al., 2013) and UC2 (Diederich et al., 2020) as well as sharing initiatives such as the Flamingo (Power and Huisken, 2019). Given these investments, there is high motivation to enable the cell and developmental biology community to utilize these tools.

With this brief overview, it is easy to appreciate that biologists seeking to newly utilize light-sheet microscopy in their scientific investigations are faced with an overwhelming number of hardware (both optical and computing) and software choices. Without a background in computer science, many biologists find existing image analysis options difficult to distinguish, let alone choose between, in part for technical reasons and in part due to hype in the fields of “accelerated computing,” “big data,” and “deep learning.” Often scientists excited about a “visualization” tool only later are disappointed to appreciate the fact that “visualization” is just being able to visually examine the raw data and may not encompass any processing/computational functionality. In institutions, departmental IT staff may or may not be aware of the unique computational needs demanded by light-sheet datasets for visualization or analysis and thus not understand the justification for the cost of high-end analysis workstations, fast network transfer, or access to on-premises and cloud computing resources. Bringing to bear biological insight from light-sheet microscopy data is such a multidisciplinary endeavor that typically no single person has a clear and comprehensive understanding of the requisite steps, creating potential pitfalls and further exacerbating this challenge. To help biologists communicate with software developers, sales representatives, IT professionals, and HPC experts about their image processing needs, we attempt to provide structure and context to relevant basic computing concepts and a process for selecting analysis tools.

## Constantly Fluctuating Landscape of Tools

By the time one has surveyed the landscape of light-sheet analysis tools it has already changed. Once the hardware is purchased, arrived, and tested, it is out of date. Software dependencies are difficult to keep compatible. While these common issues can feel overwhelming, they are not insurmountable once one is familiar with the nature of this development process. Since the light-sheet imaging and analysis landscape is a multifaceted, rapidly moving target, we believe familiarity with a few basic computing concepts will help biologists keep up with this moving target and be able to confidently provide invaluable feedback to software developers.

**FIGURE 2:**
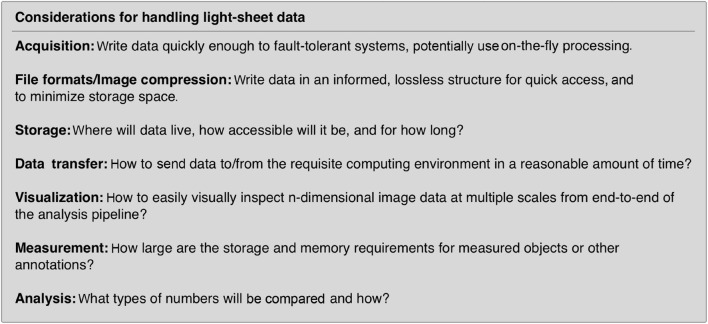
Data handling steps that require special attention with light-sheet image data. If possible, it is advantageous to select a compressed file format that can be utilized by the requisite analysis software. Unfortunately, not all analysis software can read all image formats and often data must be resaved or restructured as it travels through an analysis pipeline.

Creating a robust image analysis pipeline was already a difficult task as one had to sort through the different processing steps and order them in a way to produce the most reliable outcomes compared to ground truth annotations. However, with light-sheet data we are now confronted with the possibility that the data do not easily fit in the pipes we choose. At which point of handling light-sheet data do we need to think more carefully compared to typical confocal microscopy data sets? For most light-sheet microscopy applications, we should adapt our thinking with respect to the items listed in Figure 2. If a good strategy is laid out from end to end of this data-handling continuum, from acquisition to analysis, bottlenecks in processing and excessive data wrangling/resaving may be avoided.

## Common Data Sets and Computational Tasks for Light-Sheet Microscopy

Light-sheet data must first be acquired before any analysis can be performed, and beforehand it is useful to understand the expected size of the data, the computational environments available in a given lab or institution to process the data, and the typical components of a light-sheet data processing pipeline.

**FIGURE 3:**
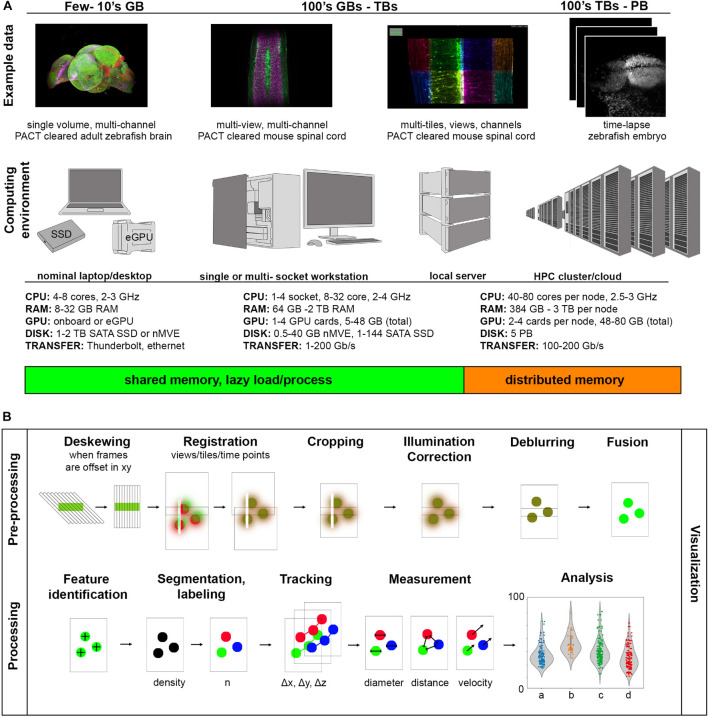
Range of light-sheet data sizes, computing environments, and processing tasks. **(A)** As data size increases, increasingly parallel computation helps to prevent bottlenecking during image analysis. **(B)** Components of a light-sheet image analysis pipeline have highly variable pre-processing steps dependent on the particular type of light-sheet microscope used and more uniform processing steps depending on the biological measurement of interest.

Raw data size can be estimated with a combined understanding of the biological system in question and the type of light-sheet imaging planned. Figure 3A shows a set of example data across a wide spectrum of sizes along with computing environments that are generally useful at a given scale. The examples we provide are 16-bit encoded, meaning each voxel takes 2 bytes of memory to store in the absence of some compression scheme, and the samples are imaged with either 5× 0.16 NA objective or 20× 1.0 NA objective on a Zeiss Z.1 light-sheet microscope. On the smaller end of the scale, we show a multi-channel data set of a cleared adult zebrafish brain imaged at relatively low resolution resulting in a dataset typically tens of gigabytes (GBs). The purpose of this experiment was to simply map the anatomical distribution of a developmentally important reporter gene in the central nervous system, and since this investigation does not require single-cell resolution, a higher-resolution data set would make visualization and analysis more difficult than necessary. The quality of the clearing is also such that only a single view is required, further reducing the data size. In general, it is desirable to try to use the minimum sampling in any dimension that can address a particular biological question, meaning higher resolution is not always better. However, if illumination is decreased across the specimen due to light scattering, or if more isotropic resolution is desirable, it is possible to utilize several image volumes acquired from different illumination angles to create a more faithful representation of the original specimen. These additional acquisitions increase the initial amount of data needing to be visualized and processed by a factor of the number of views and can begin to approach 100’s of GBs. Here we show an example of a passive clarity technique (PACT) cleared mouse spinal cord imaged from five different angles so that the views can be deconvolved and fused into a single isotropic dataset for improved tracing of spinal neural tracts. This approach increases the data size needing to be handled in pre-processing steps but for downstream processing yields comparable size data to a single volume (depending on how anisotropic the original data volumes were, and if the deblurred data is saved at higher bit-depth). For measurement of features at cellular and potentially sub-cellular scale, however, higher resolution volumes tiled across a region of interest may be acquired, increasing the data size by a factor of the number of tiles. This approach typically yields larger data sizes in both the pre-processing and the processing steps of image analysis as the datasets are both high-resolution and over a large spatial scale and can easily fall in the terabyte (TB) range. For dynamic processes, live specimens add the temporal dimension, with data size scaling according to the number of time points as shown in our example of the time-lapse of zebrafish embryonic brain development. Such datasets can approach the petabyte (PB) scale. These examples illustrate the usual fundamental dimensions of what is often referred to as “n-dimensional” imaging in light-sheet microscopy including spatial dimensions *x*, *y*, *z*, spectral channels, illumination angles (can also have different detection angles), tile numbers, and timepoints. To keep data sets of manageable size, balancing the data size in each of these dimensions is wise but also requires detailed knowledge of the biological system in question.

A computing environment is the combination of hardware and software used for a particular computing task. Figure 3A shows how these computing environments can scale to accommodate increasingly larger sizes of light-sheet image data from nominal laptops or desktops, to analysis workstations of various sizes, to local servers, to high performance computing clusters (whether on-premises or through cloud computing services). We provide a range of usual specifications of the hardware along this spectrum, though machines with specifications outside of these ranges can of course be constructed. One of the most important things to consider early on is what computational resources are available for a given project, which will guide the software approach implemented. It is usually the case that non-computer scientists find single shared memory systems easier to interface with, so there has been a strong drive to create workstations with larger shared memory that can accommodate the dataset and intermediate calculations during a processing algorithm. Such high-end workstations with TBs of shared memory can be quite expensive. Also, understanding how much memory will be required for a given analysis task, even when the data size is known, can be difficult because such algorithmic and software details are often not specified in a way that is accessible to the end user. This situation is a relic of the days when biologists could take for granted that the computational resources they were familiar with and had easy access to would be more than sufficient to process their image data. Since it is now frequently not the case that the entire data set can be loaded into computer memory, efforts have been made to create visualization and analysis tools that handle data-intensive computation by feeding smaller parts of the data to the memory at a time so as not to exceed the system memory (“lazy” load/process) or on heterogeneous distributed memory systems (that typically require more computer science expertise to interact with). In both approaches, graphics processing unit (GPU) acceleration may be employed, requiring transfer of data between system and GPU memory for external GPU boards.

In enumerating common tasks in a light-sheet image analysis pipeline in Figure 3B, the “visualization” step is drawn across all other individual “pre-processing” and “processing” tasks. It is difficult to understate how important visualization at every step of any image processing pipeline is, especially for quality control, tuning processing parameters (such as various threshold values, kernel sizes, etc.) efficiently, and to detect processing artifacts. The ability to quickly preview the data and computational results, especially when an analysis pipeline is being drafted or an existing one is applied to a new kind of image data, should be non-negotiable. The reality that most bioimage analysis pipelines, including those applied to light-sheet data sets, often need to be adapted or modified from project to project and are more frequently semi-automated than fully-automated, underscores the importance of previewing results step-by-step.

The term “pre-processing” typically refers to computational effort put toward accounting for measurement artifacts and reconstructing an even more fiducial representation of the object being imaged (that could potentially be compressed with out loss of important information). The geometric peculiarities of the type of light-sheet microscope being used, the particular imaging parameters, and the optical quality of the sample will affect the kinds of pre-processing steps one must consider. For those light-sheet acquisitions where the sample or light-sheet move relative to each other at a tilted angle to the detection objective (for some modes of lattice light-sheet microscopy, open-top light-sheet microscopes, and single objective light-sheet microscopes), the first step will typically be to deskew the data so that visualization and analysis software can interpret the voxels from each volumetric acquisition on a common global three-dimensional coordinate space. Next, if there are multiple volumetric acquisitions (different channels, angle views, tiles, or time-points), interpolation and registration algorithms will be used to put these onto a common global coordinate system. This step is necessary because of imprecision in stage coordinates, sample motion relative to the microscope stage system, and spherical and chromatic aberrations. Inclusion of fiducial markers such as fluorescent beads are often utilized to register views to each other. At this point in the pipeline, the data at a given spatial location are all still separate unique values. To reconcile these differences into a coherent single spatial representation of the data at a given time, some type of fusion algorithm is applied, often in concert with a deblurring/deconvolution step to improve image contrast and resolution if possible. Another aspect to consider, which may be done prior to, after, or in the absence of deconvolution/fusion is illumination correction. It is sometimes possible to correct for uneven illumination (such as vignetting in the case of some larger FOV acquisitions) or striping artifacts (occurring when angular spread of the illumination light is low and/or parts of the sample or other objects scatter or absorb the light, casting a shadow through the image). In the case of time-lapse data sets, photobleaching correction can be applied.

Once a reasonable representation of the object is created, the object may be registered anatomically to an existing atlas, such as the Allen Brain Atlas for the adult mouse (Lein et al., 2007), or the “processing” component of the analysis pipeline begins. Processing typically refers to computations applied to the image data to extract specific features of interest. The “extraction” can refer to providing a spatial coordinate where the feature is located (as in spot detection algorithms) or enumerating a volume in the image where a particular feature is located (as in segmentation algorithms). The type of information extracted could also be a property of the object at a certain location in space, such as identifying anisotropy of features and orientation of objects or structures in the space. In the temporal regime, it will be useful to track objects and their properties over time. If we think of our example data sets, it would be useful to segment anatomical brain regions, follow axonal tracts, segment cells and examine their morphology, or identify cell nuclei and track their movements and divisions, to name a few biologically relevant aims. It is useful at this point of the analysis pipeline to perform an assessment of the quality of the results of the computationally derived objects compared to those produced by expert annotation (Taha and Hanbury, 2015). With these types of objects we can then compute further measurements, such as the relative reporter expression in different brain regions, connectivity in different regions of the spinal cord, variability of cellular morphologies within a particular tissue, or cellular velocities. Typically these measurements are acquired for replicate groups, a control and experimental group in the simplest case, and the experiment is performed multiple times to ensure repeatability. But what sort of indication is there that the experiment is repeatable? Statistical analysis of the measured outputs is used to look at the distribution of the data sets and test for significant differences. It is certainly also desirable that when an image analysis pipeline is developed using one experimental data set and the experiment is repeated, that the same analysis pipeline can be employed without tinkering with parameters, and produce the same results. When there is confidence in the results, they may be used as parameters in computational models of biological processes or they could serve as an experimental result to compare with a theoretical result.

## The Journey of a Voxel

**FIGURE 4:**
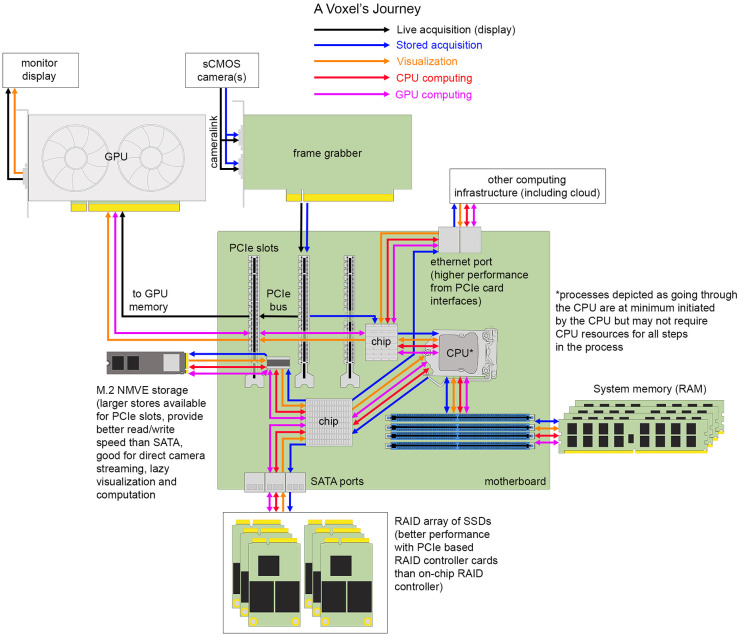
The journey of a voxel. Hardware components and interfaces for transferring data between them on one example of a typical image analysis workstation.

Having provided an overview of different scales of computing infrastructure and common image analysis pipeline components, we turn to enumerate computer hardware in finer detail. An integrated overview of basic computing hardware is shown in Figure 4. In the same way that understanding the compartments of a cell and their functions is important for thinking about different types of signal transduction pathways in cells and tissues, understanding the basic compartments of computing infrastructure is important for thinking about different pathways for scaling an image processing pipeline on an individual workstation or high performance cluster, which is still generally a unique composition for each research project involving light-sheet microscopy data.

### Camera to Peripheral Component Interconnect Express Bus

A voxel is the three-dimensional unit of a digital image that arises from the design of modern digital cameras and the physical spacing between acquired image frames. The most popular cameras for light-sheet microscopy are sCMOS cameras with low read noise (<2.0 electrons), high quantum efficiency (>80%), with sensor architectures trending toward larger, faster chip sizes for capturing larger, dynamic, diffraction-limited fields of view or for multiplexing smaller fields of view (from different channels or image planes) projected to different areas of a single chip. Back-thinned illumination for better sensitivity is increasingly desirable when read noise can remain comparably low. Other camera options include CCDs, EM-CCDs, and intensified cameras. The key overarching concept to understand is that photons hitting the discrete pixel elements of the camera sensor are transduced into electrical energy that flows under the control of electronic circuits built into the camera itself to an analog-to-digital converter (ADC). The purpose of the ADC, which in most systems is now on the camera itself, is to assign a binary-encoded value to each individual voxel proportional to the number of photons that were captured at each location. These values can be transferred to an electronics board called a frame grabber through a specific protocol, typically camera link for high-speed imaging in many light-sheet microscopes. The frame grabber has its own hardware and software that connects the device to the motherboard of a computing system with a peripheral component interconnect express (PCIe) interface and relays acquisition instructions to the camera from the acquisition software run within the operating system. An important component on the frame grabber is the direct memory address (DMA) controller that can relay image frames to GPUs to be rendered to the computer screen or for computational pre-processing, to computer memory to buffer for storage, or directly to disk without having to use central processing unit (CPU) resources for each voxel. The extent of these different DMA functionalities will depend on the design of the peripheral device and the architecture of the motherboard. Real estate of PCIe lanes is in short supply when multiple devices need to communicate on the PCIe bus, which is the electronics of the motherboard that voxels must flow through. In addition to frame grabbers, GPUs, RAID controllers, fast read/write storage, and network cards may occupy this space. Complicating matters, the total number of pins present on the PCIe slots may exceed the number of lanes available for simultaneous use, so care must be taken that components are selected to allow for near optimum performance. Devices plugged into a PCIe ×16 slot, if not provided enough lanes on the bus, will not be able to perform to specification.

### Peripheral Component Interconnect Express Bus to Central Processing Unit, Memory, Graphics Processing Unit

PCIe bus traffic is directed by the computer’s operating system with the help of the CPU and several smaller processors built into the motherboard. For light-sheet acquisitions including multiple cameras operating at high frame rates, the rate of data generation can exceed the typical write speed of many storage devices and so the data is often buffered in system memory. System memory refers to the random access memory (RAM) storage that operates only when a computer is powered on (as opposed to persistent storage that can retain values when power is off) that holds instructions and data that will be accessed directly by the CPU. Acquisition software for light-sheet microscopes can be incorporated with on-the-fly image pre-processing and processing steps that utilize GPU or CPU computational resources prior to data storage. This approach can reduce overall analysis time significantly but also runs the risk of loss of information from the raw data. The limitations of pre-processing steps that involve interpolation (e.g., deskewing) and assumption of point spread function shape (e.g., deconvolution) are still debated. Additionally, registration results are not always optimal and fusion of the data under these circumstances significantly degrades image quality. Finally, some funding and state agencies have requirements for preservation of raw data for a certain period of time, in which case one must be careful to consider what is a reasonable definition of “raw” data.

The core of a computer is the CPU which must be running a program to trigger the acquisition of frames from the microscope camera(s). Not long ago, processors contained a single computational core, often referred to as an arithmetic logic unit (ALU) that takes two binary inputs (data) and an instruction (also translated from higher-level programming languages into a binary-encoded input) and performs a rudimentary operation resulting in a binary-encoded output. These inputs are progressively moved from system memory through a series of on-processor memory stores called caches (e.g., L3, L2, and L1) until they can be loaded into the registers operated on by the ALU. More complex operations are combinations of rudimentary calculations. Modern CPUs have increased the number of cores up to tens of cores per processor, with most of these cores having multi-threading capabilities (discussed later). In this modern configuration, several software can operate in parallel as their processes can be assigned to different cores. Additionally, motherboards that support more than one CPU are now commonly used. Most of the major computer programming languages people are familiar with (e.g., C++, java, and python) are abstractions to interface a human-language computer user or programmer with the binary language of a CPU to accomplish image processing and data analysis tasks (and of course emails, gaming, etc.).

Most motherboards have an on-board GPU that prepares data to be rendered to a computer screen, but the capabilities of these GPUs can be minimal compared to the PCIe-based GPU boards that have space for more dedicated memory and GPU cores. GPU architecture is different from CPU architecture in that there are orders of magnitude more computing cores (ALUs) on a GPU. The field of computer graphics encompasses some standard operations, especially those involving manipulation of matrices (which are a common way to represent n-dimensional light-sheet data). However, giving software developers access to these functions is not accomplished through the standard computing languages but rather graphics-specific application programming interfaces (APIs) (e.g., CUDA developed by NVIDIA, OpenGL, and OpenCL). When needing to interactively display large data acquisitions or rapidly render 2D representations of a three-dimensional object, one will benefit greatly from a GPU. The larger the on-board memory of the GPU, the better it can be utilized for other computational image pre- and processing tasks as well. The data must be first loaded into the GPU’s dedicated memory, computed on, then returned to system memory. Since these memory allocation processes take time, computation is best accelerated when several computing operations are chained together before a final result is delivered back to system memory. One of the most popular types of image processing performed primarily on GPUs are the training of deep-learning networks and their use for prediction of objects and other types of image properties.

### To Data Storage

Voxels waiting in system memory will be short-lived unless they are written to one of a wide variety of long-term, non-volatile data storage devices. This writing process involves software instructions concerning where the data will be stored, how the data should be efficiently organized on disk, whether, or more likely, which compression scheme should be applied, how long the data will need to be stored, and how accessible it should be.

Generally, the farther voxels must travel, the slower the write speed will be. However, multiple factors along the way have significant impact on write performance. It is important to consider the speed of data reading (and writing) by the storage drive controller (a small computer chip that lives on the drive itself), the speed of the connectivity between the drive and the computer, the size of the drive, and the ways in which such a drive can be combined with others into a larger unit. Solid State Drives (SSDs) are typically built on a floating transistor technology called NAND flash memory and, having no mechanical moving parts like their older disk-spinning counterparts Hard Disk Drives (HDDs), are orders of magnitude faster at reading and writing data (microseconds compare to milliseconds). SSDs can connect to the motherboard using SAS or serial ATA ports (SATA), as the HDDs these ports were designed for typically do; however, the SATA interface controller is not fast enough to keep up with the data read and write speeds achievable by SSDs. To take full advantage of the speed of SSDs, faster interfaces such as non-volatile memory express (NVMe) were created that use PCIe protocols to interface with permanent storage devices. Size of the drive is often referred to as the form factor, which is relevant when configuring workstations or servers to ensure efficient use of space and heat dissipation. For workstations, motherboards will usually have one or two M.2 slots, which accommodate small SSDs that have PCIe ×2 or ×4 connectors, as well as PCIe slots for E3 form factor SSDs that have larger bandwidth and storage (PCIe ×8 or ×16). Additionally, there will be connections on the motherboard for SAS or SATA cables to transfer U.2 drives (2.5″ SSDs), though these can also be NVMe capable.

Going up from the level of the individual disk, it is important to understand how multiple disks can be utilized together to improve data read and write speed, as well as data stability, using Redundant Array of Independent Disks (RAID) controllers. Moderate performance raid controllers can be found already built-in to motherboards or such functionality provided by RAID software applications, however, it is likely the data streaming demands of light-sheet microscope acquisition will require an external RAID controller card installed into a PCIe slot. Common RAID configurations are RAID 0, RAID 1, and RAID 10. In a RAID 0 configuration, the RAID controller spreads the data across separate disks simultaneously (called striping), which parallelizes and thus speeds up data writing. While this sounds immediately useful, this approach puts the data at risk since if one drive fails, the entire data set is unrecoverable. Alternatively, in a RAID 1 configuration, the RAID controller sends the same data to all the disks (called mirroring), writing the same data on multiple drives as backups. RAID 1 protects against drive failure but provides no speed-up and decreases effective storage size by half. Both speed and redundancy, however, are design components of a RAID 10 configuration, where data is both striped and mirrored to a minimum of four drives. The effective storage of RAID 10 configurations is still cut in half, however, which is expensive. RAID 5 is a popular alternative that reduces the excess amount of space required for data redundancy by using a strategy called parity, which is a logic calculation between two bits of data striped to different disks. Rather than both bits being also mirrored, instead the and/or logic between the bits is encoded as a single bit that stored on a third disk. This strategy requires an extra calculation and care to keep the bit pairs together, but if one disk fails the missing data can be reconstructed from the parity bit that has been striped to either of the other two disks.

On-board drives are common for acquisition and short-term storage, but ultimately data is often sent to be stored on an external device such as a local storage server, HPC cluster, or the cloud for sharing, computing, and/or long-term storage. Recommendations for networked device solutions depend on available resources and data size (Andreev and Koo, 2020). Consulting with department IT or institutional HPC colleagues will help to identify the best solution given available resources, but for those labs without such support, DIY direct-attached storage [such as Just a Bunch Of Disks (JBOD)] that connect with a SAS connection may be a good option, or if the acquisition computer has a high-speed network port (10–100 Gb/s), a network-attached server can also be assembled or purchased. These longer-term, larger storage solutions are still often composed of HDDs due to cost. These external devices can then be accessed remotely, networked to other HPC resources on premises or to the cloud. One counter-intuitive point is that cloud resources are most reliable for mid-range data sizes (∼10 GB) as larger data transfers can trigger slow-downs by service providers, require specialized file transfer software, and even under the best of conditions may still result in impractical file transfer times. In the future, development of edge computing workflows that aim to minimize network travel by providing computational and storage resources to an edge device (in this case a light-sheet microscope) at the most physically proximal node of a provider network (edge node), could address some cloud service constraints. Intelligent pre-processing, compression, and/or data abstraction at such edge nodes could also limit the data size subsequently sent to cloud storage.

While many scientists that routinely use microscopy tools are familiar with the TIFF file as a reliable data storage format, this file specification is not well-suited for large image data. Given the size of most light-sheet data, the type and structure of the file the voxels will be stored in is important to consider in advance to reduce the need to perform “data wrangling,” that is, to re-save or modify the structure of the data so it can be computed on by a given analysis software and to improve data access speeds. Such considerations can also be very important for speedy data visualization. Recall in the discussion of RAID configurations that data sent to multiple disks was called “striping.” This is the case because, even though we think of image data as 2D, 3D, or ND arrays, on disk they are by default stored as a single stripe of bits ordered into a line that must be accessed sequentially. Now consider these bits are lined up on disk row-by-row so that the last voxel of one row is next to the first voxel of the next row. If one was interested to access the voxel physically below the last voxel of a row, it would be necessary to search an entire extra row. However, if the image is split into smaller 2D “chunks” and each chunk is stored row-by-row, the time to access the related voxels is reduced. Useful file formats also frequently support image “pyramids” that store the chunked multi-dimensional data at full resolution and increasingly lower resolution versions. In combination, multi-resolution chunks enable the most rapid access to spatially relevant subsets of image data. When browsing the data with visualization software that supports these file types, lower resolution data rapidly give the impression of the sample structure while the high-resolution data are quickly read and displayed, providing a real-time experience. Multi-resolution chunks can also be useful for speeding certain computations that may not require full resolution. When using commercial microscopes and software, it is often not possible to write data to a more generally open chunked pyramid file type (e.g., HDF5, zarr, OME-TIFF, and N5), but companies are increasingly utilizing these techniques along with lossless compression techniques. In addition to the values of the voxels in which we are primarily interested, it is usually the case that important metadata about the microscope and camera settings are stored with the image data and keeping these pieces of information together is an important aspect of scientific reproducibility. Unfortunately, there is not a single consensus on the overall best file structure and therefore some conversion and data wrangling is likely (Moore et al., 2021).

### Out of Storage for Processing

Having seen the path that a voxel must traverse to be stored, one has already seen the relevant paths that would be traversed for additional processing. Data must be available in system memory to be operated on, so the data will be loaded from storage to RAM. Then, if the computation is to be carried out on the CPU, the data will be sent to the processor as requested, or if the computation is to be carried out on the GPU, the data will travel on the bus from system memory to the GPU’s dedicated memory and operated on by the GPU. Computational results are temporarily stored as variables in either system memory or GPU memory and at some point, written back to permanent storage. One important thing to consider when putting together an image processing pipeline is to try to minimize the effort put toward transferring data and only do so (for example load the data into GPU memory) if the speed-up in computation outweighs the data transfer time. Another key consideration is whether the data fits in the relevant memory storage space and if not, what can be done to split the data into usable chunks for a given processing task.

Computing hardware and software changes have been slow and steady but could change drastically in the near future, creating an ever-bigger challenge requiring more computing literacy and even better communication across disciplines. We hope this brief overview of computing hardware is empowering to biologists and microscopists so they can consider the steps of designing and implementing demanding image processing workflows in a more concrete and less abstract way.

## What Can Parallel Computing Mean?

We have observed that scientists who are unfamiliar with larger data intensive computing workloads often assume that computational hardware resources available will be automatically utilized by a given software. However, as discussed in the previous section, data that is to be processed must be in an accessible location to the given computing hardware, whether CPU or GPU cores, and these directions must be explicit. If multiple nodes of a computational cluster are to be utilized, the software also has to make this explicit. Thus, it is regrettably possible to select software that fails to take advantage of all available computing resources, especially when hoping to scale across a distributed memory system.

**FIGURE 5:**
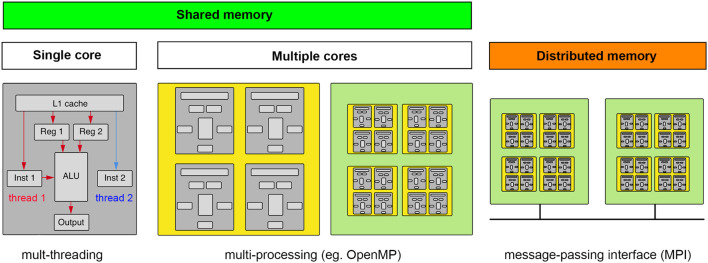
Computing concepts for scaling up and scaling out. Computation requires data and instructions to be loaded into registers directly accessible by an arithmetic logic unit (ALU). Multi-threading makes computations parallel by taking advantage of dead time when data are being fetched. Multi-processing is when the computation can be spread to multiple cores (ALUs), whether on a CPU or GPU, that have access to the same memory (GPU’s typically having their own smaller on-board memory). If the data and/or computations do not fit in shared memory, a message-passing interface must help coordinate the broadcasting of data and computations across a distributed memory system. (gray, core; yellow, CPU; green, motherboard).

Parallel computing is often a good solution for light-sheet image analysis, but to best utilize the power of parallel computing one must appreciate the different types of hardware approaches to parallel computing and the algorithmic nature of a particular task-specific parallel computing problem. The former is shown in Figure 5. One approach to parallel computing, called multi-threading, takes advantage of the extra time required to retrieve data and deliver it to a cache to await a particular computing instruction. On a given computing core, instructions are threaded through the ALU as data become available. It is worth noting that this is not truly parallel computing. Multi-threading is programmed at the level of the standard libraries of a given software language that will most likely relate to a C++ compiled code. The next level of parallelism is to compute on multiple cores on a given processor chip, which is typically referred to as multi-processing and relies on particular software libraries that direct data and instructions to different cores of one or more processor chips on a single mother board. For certain types of computations, using the cores of a GPU is highly advantageous but requires the data be sent to the GPU’s on-board memory which may be significantly limited compared to the overall system memory. Light-sheet data thus typically requires some cropping, compression, or thoughtful chunking to be processed on a GPU. Finally, if even more computational power is required, a “master” computer will pass instructions and data to different computing nodes, each operating as independent computers with their own isolated memory, which will execute computations that will be sent back to be compiled by the master node. The “nodes” can be comprised of CPUs and GPUs depending on the nature of the computational task.

It is easiest to implement parallel computing when the nature of the computational step is “embarrassingly parallel,” meaning the data can be cleanly and arbitrarily split into convenient sizes to spread across available computational cores and/or nodes. An example of this type of parallel problem would be having many timepoints in a dataset that are each small enough to be handled by a single node. Instances of the image analysis software can be created on as many nodes as available, in the case there are as many nodes as timepoints one could process each time point simultaneously. However, the spatial dimensions of many light-sheet data volumes make it possible that a single time point may not fit on a single workstation or computational node. In this case, one must divide the data into chunks that can fit on a given node (this will also sometimes be the case for fitting data into GPU memory). While the chunk size that is specified by the file structure of the data may be a natural choice, this is not always the case. We again must assess the nature of the parallel problem. For example, convolutional filters that are commonly used in image analysis calculate a new voxel value using voxels surrounding the voxel in question, meaning that the chunks distributed across available nodes or to GPU memory must be overlapping and in some cases how to best recombine the spatial volume is unclear. The overlap has the effect of increasing the overall data size, but if these already exist from tiled acquisition they could be useful.

## Evolution of Computing Environments for Light-Sheet Image Visualization and Analysis

Computing hardware components have the potential to be utilized in a light-sheet image processing workflow provided the communication between the parts is supported by the requisite software. Table 1 provides an overview of the hardware/software solutions employed by several research groups referred to in Figure 1, where such details are available in the literature. From these specifications, one can see that light-sheet image processing has evolved over time from computations executed on a single thread of a single CPU core to computations that can be scheduled in parallel to multiple multi-threaded CPU cores and GPU cores on a single or across several computer nodes. Storage solutions have shifted from uncompressed TIFF files on standard HDDs to multi-resolution chunked pyramid file types with lossless compression on SSDs. These examples are from academic research, but commercial light-sheet acquisition software (e.g., Zeiss Zen) and other image analysis softwares (Imaris, Vision4D, and Amira) have similarly been adapting their computing environments to accommodate larger image data files.

**TABLE 1:** Sampling of light-sheet computing environments.

Paper	Data size	Data storage	CPU resources	RAM	GPU resources	Software	File Type	Performance (volume <5 GB)
Huisken et al., 2004	<100 GB	–	1.8 GHz, single-core	–	–	Matlab 6.1		fusing one volume took 24 h
Swoger et al., 2007	–	–	2.8 GHz, dual-core			Matlab 7.0.4, C#		Weiner MVD on one volume overnight
	–	–	10 CPU cluster	20 GB		Python		MAPPG MVD on one volume in 1 h
Keller et al., 2008	3.5 TB	2 RAID 0 (6 TB total storage)	5000 CPU cluster	–	–	Matlab		48 h to track single time-lapse
Preibisch et al., 2010	–	–	2.8 GHz, quad-core	64 GB	–	Java/FIJI		constellation bead-based registration in 2.5 min
Planchon et al., 2011	–	–	–	–	–	Amira 5.3		iterative max liklihood deconv. in 12–15 iterations
Truong et al., 2011	–	–	–	–	–	Matlab, Imaris		–
Tomer et al., 2012	<100 TB	100 TB SATA drives (separate server)	2x 3.3 GHz, 6-core, 12-thread	96 GB	Quadro FX 5800, 4GB	Matlab R2011b, C++		single timepoint fused on a multi-threading core in 180 s
								up to 12 time points in parallel across 12 cores
Wu et al., 2011	~260 GB	–	–	–	–	Matlab, StarryNite, AceTree		semi-automated C.Elegans lineage tracing
						DeconvolutionLab (ImageJ)		
Wu et al., 2013		–	2.4 GHz, 6-core, 12-thread	6 GB	–	MIPAV, python, matlab		registration, joint deconvolution of 1000 volumes in 7 h
Kumar et al., 2014	–	2 TB SATA drives	2x 2.3 GHz, 6-core, 12-thread	64 GB	Quadro K5000, 4 GB	ImageJ, MIPAV		joint deconvolution 6 volumes/minute
Chen et al., 2014		–	2x 3.33 GHz, 6-core, 12-thread	96 GB	GeForce GTX TITAN, 6 GB	Matlab, CUDA, ImageJ, Amira		deskew, deconvolution, photobleach correction
Preibisch et al., 2014	GB-TB	–	4 node cluster	128 GB	2x Quadro 4000, 2 GB	Java/FIJI, CUDA	.xml/HDF5	Bayesian joint multiview deconvolution
			2x 2.7 GHz, 8-core, 16-thread		4x Tesla			depending on implementation, 1 volume in ≤15 min
Bouchard et al., 2015	–	–	–	–	–	Matlab, Amira		–
Pietzsch et al., 2015	up to 60 GB	750 GB SSD	2.8 GHz, 8-core, 16-thread	16 GB	–	Java 1.6/FIJI	.xml/HDF5	convert 60 GB to .xml/.HDF5 in less than an hour
Royer et al., 2016	–	1 TB SSD, 12 TB HDD	2x 3.1 GHz, 8-core, 16-thread	256 GB				autopilot control, image quality estimations
Liu et al., 2018	3 TB/h	–	cluster, 16-32 cores	120-240 GB	presumed	Matlab, CUDA, FIJI, ITK		deskew, deconvolution, illumination correction,
			per node	per node		ITK-SNAP, Amira, Imaris, Aivia		segmentation of cell, nucleus, and trans-Gogli apparatus
						u-track		cell tracking
Hörl et al., 2019	GB->2TB	RAID 0 SSD	2x 3.2 GHz, 8 core, 16-thread	512 GB	variable	Java/FIJI, CUDA	.xml/HDF5	stitching, fusion of 300 Gb in <9 h
Glaser et al., 2019	~ 1 TB	512 GB M.2, 16 TB SSD, 96 TB HDD	2x 3.2 GHz, 8-core, 16-thread	384 GB	Titan XP, 12 GB	python, BigStitcher,	.xml/HDF5 w/B3D compression, .tiff	1 TB of tiles fused and re-saved 12–24 h
					Quadro P6000, 24 GB	Aivia, Imaris		
Voleti et al., 2019	GB-TB	–	–	–	–	Matlab, BigStitcher, TrackPy	16-bit .tiff	registration, deskew, stitching
Haase et al., 2020	200 MB x 300	–	1.9 GHz, 4-core, 8-thread	–	Intel UHD 6230	FIJI/CLIJ, OpenCL		speed-up of common analysis tasks 2-188X
			2x 2.1 GHz, 8-core, 16-thread	–	Quadro P6000, 24 GB			
Chang et al., 2019	–	2 TB SSD	3.1 GHz, 10-core, 20-thread	128 GB	–	FIJI, Matlab 2017a	OME-TIFF	deconvolution, shearing
Sapoznik et al., 2020	–	16 TB SSD RAID 0	2x 2.2 GHz, 8-core, 16-thread	128 GB	Titan RTX, 24 GB	python (Numpy, Numba)		deskewing, deconvolution on a volume in 125 s
						BigStitcher		

One area that highlights the special difficulties with light-sheet microscopy data is that of multiview image reconstruction. As we have emphasized, there are frequently multiple views of the sample in question that could theoretically be combined in any number of ways. What is especially interesting to biologists is the possibility of increasing the spatial resolution of the resulting image by utilizing information coming from views with complementary spatial frequency information. Computational work toward this goal preceded the first SPIM microscope in a successful attempt to improve widefield fluorescence microscopy resolution by acquisition and fusion of multiple views (Swoger et al., 2003). This work was extended to include deconvolution (Swoger et al., 2007). Deconvolution is a signal processing concept recognizing that any measurement of an object by an instrument is the convolution of the object with the instrument’s impulse response. Thus, if one has a measurement of a given instrument’s impulse response, one can attempt to computationally recover a higher-resolution version of the object in question by deconvolving the measurement with the impulse response. In the case of a fluorescence microscope, the impulse response is the microscope’s point spread function (PSF) which can be theoretically computed and experimentally measured (often from the fluorescent beads embedded with the sample in agarose for fiducial markers) and has its Fourier transform produced counterpart in the frequency domain as the optical transfer function (OTF). This fact is of interest as a convolution in the spatial domain becomes multiplication in the frequency domain, so operations are often less computationally expensive in the frequency domain despite the requisite Fourier transform. A variety of different deconvolution algorithms take different approaches to estimate the underlying image in the spatial domain or frequency domain, often as an iterative process with or without the PSF (as in blind deconvolution algorithms) (Sibarita, 2005).

There have been many efforts to apply these different deconvolution algorithms to light-sheet microscopy data, the simplest of these being ones that deconvolve the individual views prior to fusing the images together. However, due to the computational cost of this approach, even with GPU acceleration, more effort has been made to adapt the iterative steps in these different approaches to incorporate the information from different views in a single joint deconvolution. The Richardson-Lucy (R-L) algorithm has been adapted to switch between one of two orthogonal views as it iterates progressively toward a maximum likelihood estimation of the underlying image, which is implemented in a combination of MATLAB and python (Wu et al., 2013). Using an unmatched back projector (the function that maps from the measurement to the underlying object) was shown to produce similar results in tenfold fewer iterations (Guo et al., 2020). R-L has also been adapted to a Bayesian/Probabilistic algorithm implemented as a FIJI plugin that can reduce computation time on a CPU by two orders of magnitude when 5 or more views are considered in the estimation (Preibisch et al., 2014). A clever plane-wise deconvolution algorithm allows more efficient GPU acceleration (Schmid and Huisken, 2015). In all implementations, using the views jointly for deconvolution appears to provide superior reconstruction. Whether a more accurate space-variant PSF algorithm would be useful is unclear. Several reports implement spatially varying PSFs that typically are theoretically modeled based on the species of the light-sheet microscope (as the PSF calculation depends on the optics of both the illumination and the detection paths) (Temerinac-Ott et al., 2011; Chen et al., 2018), but these improvements appear to be modest (Becker et al., 2019).

Deep-learning neural networks (DNNs) trained on traditionally deconvolved images have also been used to infer the underlying object in an image (Weigert et al., 2018; Bai et al., 2019; Guo et al., 2020). Once trained, such networks can use basic linear algebra operations to quickly predict a desired outcome. Care must be taken to validate such approaches, however, since training data sets for such models are never comprehensive. Another appealing aspect of this approach, in addition to incomparable computational speed, is that it may no longer be necessary to embed fluorescent beads with specimens (provided a performant non-bead-based registration algorithm is available), which is desirable as it can be difficult to find compatible fluorescent beads for certain clearing solutions and the beads often must be computationally extracted for other visualization and image processing steps.

Deep learning techniques are increasingly popular for light-sheet image analysis and frequently implemented as python scripts utilizing libraries that build on GPU APIs (e.g., PyTorch and TensorFlow). Some examples include detecting bacteria in larval zebrafish intestine with 3D convolutional neural network (CNN) (Hay and Parthasarathy, 2018), puncta segmentation in sub-cellular lattice light-sheet microscopy volumes with 3D-UNET architecture (Schoneberg et al., 2019), high-content screening of mitotic phenotypes in spheroid cultures using diSPIM and deep learning (Eismann et al., 2020), and Deep-SLAM, an add-on device for inverted microscopes for light-sheet imaging and DNN deblurring (Zhao et al., 2020).

## Tool Selection Process and Learning to Draft and Test Image Analysis Pipelines

**FIGURE 6:**
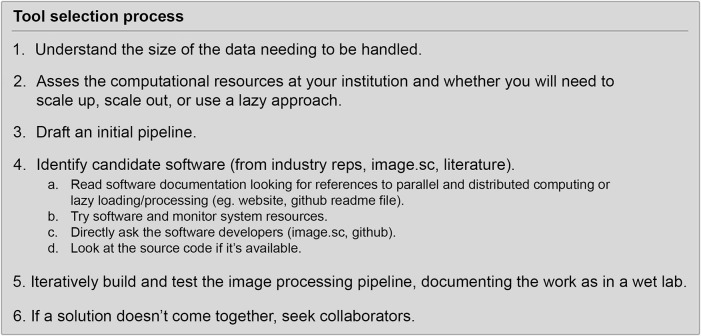
Outline of software tool selection process.

Having discussed the relevant concepts, we now present an outline to follow when drafting and testing light-sheet image analysis pipelines shown in Figure 6. The first step in the tool selection process is to understand the size of data that will be generated and to what extent the data can be cropped or compressed without loss of detail relevant to the biological question at hand. The next step is to survey the computational resources that are available and connect with the personnel responsible for maintenance of those resources (IT staff and HPC staff). With information about the limits of computational resources and data size, it is possible to predict if software capable of lazy loading/processing will be required. It is good, if one has familiarity with typical image analysis pipelines, to draft an initial image analysis pipeline with theoretical steps and without committing to any particular software or algorithm. Once the general steps are enumerated from end-to-end, one can search for candidate softwares that can handle the entire pipeline from end-to-end, or more likely, to identify a handful of software packages that best address different components of the pipeline and minimize the amount of data resaving or data wrangling required. In this endeavor, it is useful to pay attention to the quality of customer service if one is searching the commercial software space and assess the developmental trajectory of a given software (is it actively maintained, used by a variety of similar researchers) if searching the open software space. If time allows, it can be beneficial to construct several candidate pipelines to test side-by-side. During this drafting, it will benefit one greatly to keep notes in a lab book or some other documentation on the details of different software, where they are available, how to overcome any installation issues experienced, available details of algorithmic processes and computational performance, specific parameters and step-by-step execution of processing. Ideally during this testing, a manually annotated ground truth is available to quantify the accuracy of a pipeline under slightly different configurations (order of operations) or with different parameters. Without rigorous note-taking, as one would maintain in the wet lab to go back and refer to for troubleshooting, informing future experiments, and communicating results, it is easy to lose track of what has already been tried for a given computational task. Worse, one may strike on a satisfactory pipeline and combination of parameters but fail to record what was the exact implementation that gave such accurate results. Once a reasonable result is produced, write a protocol. Depending on the makeup of a given research group that seeks to utilize light-sheet microscopy data, it is also useful to consider whether collaboration is a more viable option in the case no one in the group is interested in developing the expertise required for light-sheet image analysis. Increasingly more computer scientists are drawn to the specific computational challenges associated with big image data and collaborations with them could lead to interesting new advances. Increasingly more core facilities are harboring light-sheet microscopes and the researchers there may be able to assist on-boarding students, post-docs, and investigators to the field or serve as collaborators themselves.

## Discussion

We have presented a high-level overview of computing concepts we find relevant to navigating the existing software available for analysis of light-sheet microscopy data. More detailed information is easily available online and in the cited literature. In reviewing these concepts, we have also discussed the progression of light-sheet microscopy development from optical hardware to computing hardware and analysis software.

We also want to point out some features to strive for when designing future software solutions, from the end-user’s perspective. Many biologists would say it is desirable to have analysis tools that work quickly and easily. This statement is often related to difficulties encountered when installing open source software and difficulties with learning the scope of functionality of a given software that may or may not include some basic scripting/programming. In the recent past, efforts toward providing better support for open source software through receptive developers patiently helping scientists wanting to use their software have been increasing (see image.sc forum), as well as efforts made to create better software documentation and tutorial videos. Communities like the NEUBIAS group, with their seminars broadcast online now, as well as conferences like the Images 2 Knowledge Janelia conference that went virtual during the pandemic, have made quality tutorials more accessible. We hope such efforts will continue and will make it easier for scientists to use and provide feedback to active software development teams. While on one hand end-users can be overwhelmed with too many customizable options and functionality, it is also very powerful to be able to tune parameters interactively with tightly coupled computation and visualization [e.g., FIJI’s CLIJ assistant (Haase et al., 2021)]. Having the ability to access/write data stores from a variety of software packages without resaving data would be tremendously useful. Currently, depending on the format and size of the existing data, a search for software solutions is usually confined to those that can handle the data in its existing form. Software that can enumerate memory needs clearly in advance (such as BigStitcher does for some functions) and potentially detect available computing hardware and automatically scale computation up as needed would be very powerful. Alternatively, acquisition systems that can incorporate more processing steps on-the-fly, provided they are clearly described and accepted by the scientific community, could reduce the image and data analysis bottleneck. Rather than collecting a dataset that takes years to analyze, analysis could be finished the same day. Having more application context from instrument and software developers and less hype, with software benchmarked against existing techniques with a variety of standard data sets is also desirable.

From the perspective of developers, it is helpful to enumerate these computational concepts to drive better appreciation among biologists for the need to value software maintenance and to invest in proper analysis pipeline development. Biologists can value these endeavors by citing open-source software, hiring bioimage analysts and including them as authors on papers, and advocating to funding agencies and institutions for support for software maintenance. While we have focused mostly on the world of open-source software solutions for light-sheet image analysis, it will be important to better engage the commercial sector so that more light-sheet specific analysis functions can be incorporated in powerful analysis environments like Imaris and Arivis. It would also be helpful for commercial software to be more compatible with HPC systems.

**FIGURE 7:**
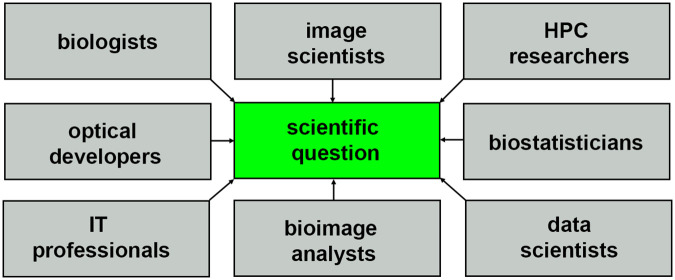
Community of researchers that help to turn light-sheet image data into scientific insight.

The nature of HPC systems will continue to evolve to support composable virtual machines and software containers, driving the need for more diverse communities of researchers with the right expertise to extract biological insights from light-sheet data sets, as shown in Figure 7. Incentives for collaborative multidisciplinary research will require more holistic storytelling, and a scientific culture that is more conscientious about sharing credit so that all parties can be equally invested in a multidisciplinary question. Consider how the story of light-sheet microscopy (Siedentopf and Zsigmondy, 1902; Néculcéa, 1903) is told, often focusing on just one name, Zsigmondy, or Siedentopf, chemist and optical physicists, respectively, depending on which field the report is from Masters (2020). Multi-disciplinary science will be benefited by increased tolerance for more nuance and complexity where recognition of scientific contributions is concerned, hopefully resulting in better communication and quicker time to biological insight.

## Author Contributions

HG and KM devised the figures. HG wrote the initial manuscript and performed all imaging. HG, SMM, NH, AP, SV, SS, AM, AY, AL, DM, KM, and LP provided revisions based on their different expertise. HG, SWM, and AV prepared samples. All authors contributed to the article and approved the submitted version.

## Conflict of Interest

The authors declare that the research was conducted in the absence of any commercial or financial relationships that could be construed as a potential conflict of interest.

## Publisher’s Note

All claims expressed in this article are solely those of the authors and do not necessarily represent those of their affiliated organizations, or those of the publisher, the editors and the reviewers. Any product that may be evaluated in this article, or claim that may be made by its manufacturer, is not guaranteed or endorsed by the publisher.
